# Blue-green tunable color of Ce^3+^/Tb^3+^ coactivated NaBa_3_La_3_Si_6_O_20_ phosphor via energy transfer

**DOI:** 10.1038/srep33283

**Published:** 2016-09-15

**Authors:** Zhen Jia, Mingjun Xia

**Affiliations:** 1 Key Laboratory of Coordination Chemistry and Functional Materials in Universities of Shandong, Dezhou University, Dezhou 253023, PR China; 2 Beijing Center for Crystal Research and Development, Key Laboratory of Functional Crystals and Laser Technology, Technical Institute of Physics and Chemistry, Chinese Academy of Sciences, Beijing 100190, PR China; 3 University of Chinese Academy of Sciences, Beijing 100049, PR China

## Abstract

A series of color tunable phosphors NaBa_3_La_3_Si_6_O_20_:Ce^3+^, Tb^3+^ were synthesized via the high-temperature solid-state method. NaBa_3_La_3_Si_6_O_20_ crystallizes in noncentrosymmetric space group *A*ma2 with the cell parameters of a = 14.9226(4) Å, b = 24.5215(5) Å and c = 5.6241(2) Å by the Rietveld refinement method. The Ce^3+^ ions doped NaBa_3_La_3_Si_6_O_20_ phosphors have a strong absorption band from 260 to 360 nm and show near ultraviolet emission light centered at 378 nm. The Ce^3+^ and Tb^3+^ ions coactivated phosphors exhibit color tunable emission light from deep blue to green by adjusting the concentration of the Tb^3+^ ions. An energy transfer of Ce^3+^ → Tb^3+^ investigated by the photoluminescence properties and lifetime decay, is demonstrated to be dipole–quadrupole interaction. These results indicate the NaBa_3_La_3_Si_6_O_20_:Ce^3+^, Tb^3+^ phosphors can be considered as potential candidates for blue-green components for white light emitting diodes.

By virtue of the special merits of high brightness, energy-efficient, life-durable, and environmentally friendly, the white light emitting diodes (w-LEDs) made from blue or near-ultraviolet (*n*-UV) emitting LEDs chips coated with phosphors have the potential to overtake incandescent and fluorescent lighting types^1^,^2^,^3^,^4^. In 1996, the w-LEDs fabricated from the blue-emitting InGaN LED chips combined with the yellow-emitting phosphors (YAG:Ce^3+^) were commercialized^5^. Unfortunately, this technology has the following problems: low color rendering index due to two-color mixing, and low color reproducibility due to the strong dependence of white color purity on the quality of phosphors^6^,^7^,^8^,^9^,^10^,^11^. To solve these problems, the w-LEDs had been fabricated employing blue, green and red emitting phosphors excited by a blue or *n*-UV chip^12^,^13^,^14^. However, the strong reabsorption of blue light by red and green phosphors reduces the luminescence efficiency in this system^15^,^16^. To overcome these disadvantages, vigorous attentions were received to exploit the emission-tunable phosphors with strong absorption in *n*-UV region^17^,^18^. Simultaneously, an energy transfer can obviously improve the luminescent efficiency and color reproducibility as well as widen the emission spectra of phosphors.

After years of efforts, a series of promising phosphors had been developed, such as fluorides^19^, silicates^20^,^21^, phosphates^12^, orthovanadates^22^,^23^, borates^24^,^25^, tungstates/molybdates^26^,^27^, nitrides^28^,^29^, aluminates^30^,^31^,^32^, etc. Among them, the silicate compounds as luminescent hosts were intensively studied because of their remarkable stability of physical and chemical properties, flexible crystal structures and relatively easy preparation process. Among rare earth ions, the Tb^3+^ ion is the best candidate for green component due to its predominant ^5^D_4_–^7^F_5_ transitions peaking at around 545 nm^33^. However, the electric dipole transitions within the 4*f* configurations of the Tb^3+^ ion is spin forbidden, resulting in the weak intensity of its absorption in the *n*-UV region and the narrow width. Thus, a suitable sensitizer is always necessary for the phosphors activated by the Tb^3+^ ion. It is well known that the Ce^3+^ ion is an excellent sensitizer transferring a part of its energy to an activator such as the Tb^3+^ ion depending on its lowest 5d state and broad absorption and emission bands from the allowed 4f → 5d transitions^34^. In this work, we reported a novel silicate host phosphor, NaBa_3_La_3_Si_6_O_20_:Ce^3+^, Tb^3+^ for the excitation by a *n*-UV LED chip, and the crystal structure, luminescent properties and energy transfer mechanism between the Ce^3+^ ion and the Tb^3+^ ion had been thoroughly investigated.

## Results and Discussion

### Crystal structure and phase formation

**Figure 1:**
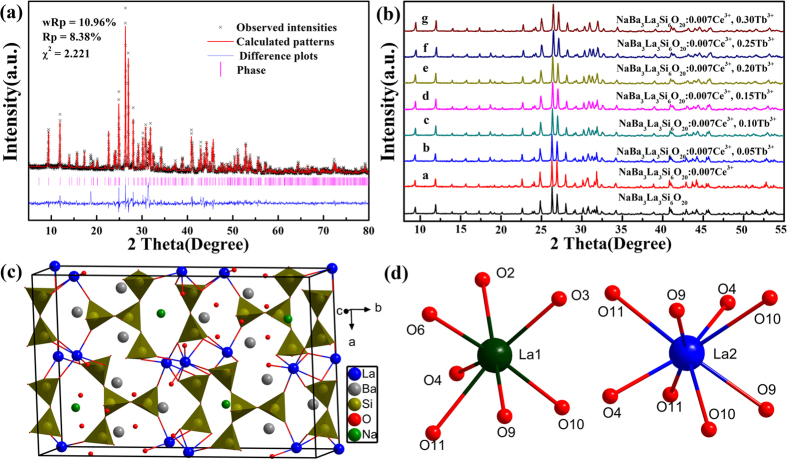
(**a**) The XRD profiles for the Rietveld refinement of NaBa_3_La_3_Si_6_O_20_. (**b**) The XRD patterns of the NaBa_3_La_3_Si_6_O_20_, NaBa_3_La_3_Si_6_O_20_:0.007Ce^3+^ and NaBa_3_La_3_Si_6_O_20_:0.007Ce^3+^, yTb^3+^ phosphors. (**c**) The structure of unit cell of NaBa_3_La_3_Si_6_O_20_ along the c axis. (**d**) The coordination environments of La1 and La2 in NaBa_3_La_3_Si_6_O_20_.

Figure 1(a) demonstrates the observed and calculated XRD patterns as well as their difference for the Rietveld refinement of NaBa_3_La_3_Si_6_O_20_. In the refinement, an initial structure model and atomic positions of NaBa_3_Eu_3_Si_6_O_20_ were adopted for the structure refinement^35^,^36^. NaBa_3_La_3_Si_6_O_20_ crystallizes in the noncentrosymmetric space group *A*ma2 and unit cell parameters are obtained as a = 14.9226(4) Å, b = 24.5215(5) Å and c = 5.6241(2) Å, which are slightly larger than those of NaBa_3_Eu_3_Si_6_O_20_ due to large ionic radius of La^3+^ ion^37^. As shown in Fig. 1(c), the basic structural units are distorted (SiO_4_)^4−^ tetrahedra which are further linked by the Ba, La, and Na atoms to build a complex three-dimensional framework. The Na atoms which are surrounded by six oxygen adopt distorted pentagonal-pyramidal geometry, the Ba1 and Ba2 atoms coordinated to seven and eight oxygen are in distorted trigonal prism and cube configuration. In the structure of NaBa_3_La_3_Si_6_O_20_, there are two kinds of La sites, implying that there are two possible types of Ce^3+^ ions in the NaBa_3_La_3_Si_6_O_20_:Ce^3+^ samples. The La1 atoms are coordinated to seven oxygen atoms to form pentagonal bipyramid while the La2 surrounded by eight oxygen atoms are in square anti-prism environment (Fig. 1(d)).

Figure 1(b) shows the selected XRD patterns of the as-synthesized representative samples of NaBa_3_La_3_Si_6_O_20_ and NaBa_3_La_3_Si_6_O_20_:0.007Ce^3+^, *y*Tb^3+^ (0 ≤ *y* ≤ 0.30) and the quantitative analysis of all the samples illustrate that the doping of Ce^3+^ or/and Tb^3+^ are successful (Supplementary Table S1). Also, it can be seen that all the diffraction peaks of the selected phosphors match well with the NaBa_3_La_3_Si_6_O_20_ phase. Even at high doping concentration of the Tb^3+^ ion (30%), the XRD patterns of phosphors are almost same with that of undoped phase, which illustrates the excellent stability and accommodation capacity for doped ions of crystal structure of the NaBa_3_La_3_Si_6_O_20_ host. The XRD profiles for the Rietveld refinement of the single element doped and co-doped samples and the coordination, occupancy and isotropic displacement parameter for all samples are listed (Supplementary Figs S1–S7, Tables S2–S9).

### Photoluminescence properties and energy transfer

**Figure 2:**
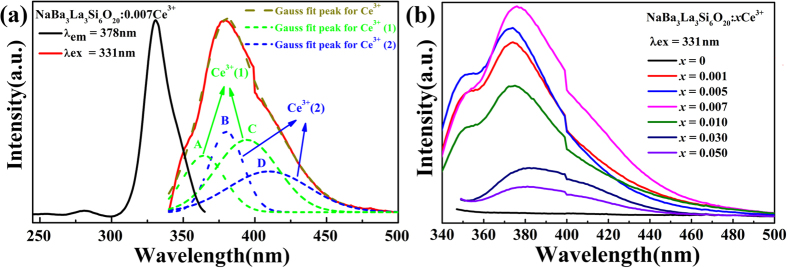
(**a**) The PLE (dark solid line) and PL spectra (red solid line) of the NaBa_3_La_3_Si_6_O_20_:0.007Ce^3+^ sample and the Gaussian peaks fitting (the green dashed lines of the Ce^3+^ (1) and the blue dashed lines of the Ce^3+^ (2)). (**b**) The PL spectra of the NaBa_3_La_3_Si_6_O_20_:*x*Ce^3+^ samples with varying concentration of the Ce^3+^ ions.

As shown in Fig. 2(a), the PLE spectra of the NaBa_3_La_3_Si_6_O_20_:0.007Ce^3+^ sample consist of three absorption bands centered at around 254, 282 and 331 nm, which arise from the electronic transitions between the ground state (^2^F_5/2_ and ^2^F_7/2_) and the levels of 5*d* excited split by crystal field of the Ce^3+^ ion^38^. Under the excitation wavelength of 331 nm, the Ce^3+^ ion doped NaBa_3_La_3_Si_6_O_20_ sample shows an asymmetric emission band extending from 340 to 500 nm with the maximum at 378 nm, indicating a possible spectral overlap originating from different luminescence centers. It is obvious that one type of Ce^3+^ ions gives rise to two emission band due to the transitions from the lowest 5*d* excited states to two ground states (^2^F_7/2_ and ^2^F_5/2_) respectively^39^. However, the emission band of the NaBa_3_La_3_Si_6_O_20_:0.007Ce^3+^ sample can be decomposed into four Gaussian components A–D peaking at 364, 380, 394 and 410 nm with the energy gaps between A and C is 2092 cm^−1^, that of B and D is 1926 cm^−1^, which are close to the theoretical value of 2000 cm^−1^ 40,41. These results imply that there should be two kinds of Ce^3+^ ions, which is consistent with the previous investigation on the crystal structure that there are two kinds of different chemical environment of La^3+^ ions in the NaBa_3_La_3_Si_6_O_20_ host.

As given in Fig. 2(b), the PL intensity of the NaBa_3_La_3_Si_6_O_20_:*x*Ce^3+^ samples increases gradually with the increase of the doping concentration of the Ce^3+^ ions and reaches the maximum when the *x* value is 0.007, and then begins to decrease due to concentration quenching^42^. It is also indicated that the Ce^3+^ ion is a sensitizer for the Tb^3+^ ion and an energy transfer of Ce^3+^ → Tb^3+^ is crucial to enhance green emission of the Tb^3+^ ion and achieve color tunable emission light. Therefore, the optimal concentration of the Ce^3+^ ion in the NaBa_3_La_3_Si_6_O_20_:*x*Ce^3+^ samples is confirmed to be 0.007.

Generally, the critical distance R_C_ between the Ce^3+^ ions can be calculated with the following equation given by Blasse^43^:


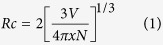


where *V* is the volume of unit cell, *x* is the critical concentration of doped ions, where the emission intensity of phosphors reaches the maximum, *N* is the number of host cations per unit cell. For the NaBa_3_La_3_Si_6_O_20_:0.007Ce^3+^ sample, *N* = 12, *V* = 2057.989 Å^3^, *R*_*C*_ is calculated to be about 25.00 Å. Dexter noted a non-radiative energy transfer usually was attributed to exchange or multipole – multipole interaction in oxide phosphors and the exchange interaction was valid only when the *Rc* was shorter than 5 Å^44^. In consequence, the concentration quenching mechanism of the Ce^3+^ ions in the NaBa_3_La_3_Si_6_O_20_:*x*Ce^3+^ samples is dominated by the multipole – multipole interaction.

**Figure 3:**
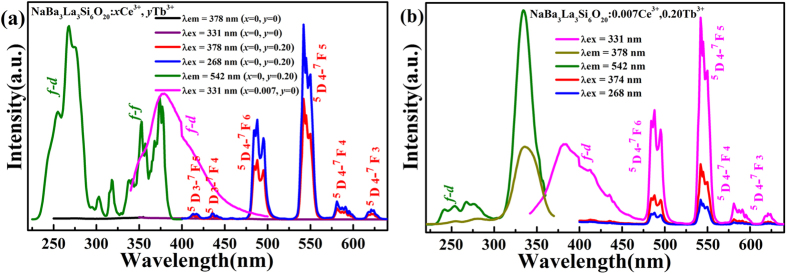
The PLE and PL spectra of the NaBa_3_La_3_Si_6_O_20_:*x*Ce^3+^, *y*Tb^3+^ phosphors (**a**), and the NaBa_3_La_3_Si_6_O_20_:0.007Ce^3+^, 0.20Tb^3+^ phosphor (**b**).

Figure 3(a) depicts the PLE and PL spectra of the NaBa_3_La_3_Si_6_O_20_:0.20Tb^3+^ sample. The PLE spectrum monitored at 542 nm exhibits a broad absorption band centered at 268 nm from 200 to 300 nm and several peaks within the scope of 300 to 400 nm. The former excited peak is ascribed to 4*f*^ 8^–4*f*^ 7^5*d* transition of the Tb^3+^ ion, while the latter peaks are from the intra-4*f*^ 8^ transitions^45^,^46^. Under the excitation wavelength of 268 nm or 378 nm, the NaBa_3_La_3_Si_6_O_20_:0.20 Tb^3+^ sample emits green light with main peaks at 412, 435, 457, 488, 542, 581 and 622 nm, which can be ascribed to the ^5^D_4_–^7^F_*J*_ (*J* = 6, 5, 4 and 3) transitions of the Tb^3+^ ion. However, because the *f*–*f* absorption is a forbidden transition, only some narrow *f*–*f* transition lines locate in the excitation range of *n*-UV LED in spite of difficultly bumping the Tb^3+^ ion^47^. There is an overlap between the emission band (magenta line in Fig. 3(a)) of the Ce^3+^ ions and the *f*–*f* transition (olive line in Fig. 3(a)) absorption band of the Tb^3+^ ions, therefore, it is potential that the Ce^3+^ ions can be sensitizers to transfer energy to the Tb^3+^ ions to enhance their absorption. As shown in Fig. 3(b), the PL spectrum of the NaBa_3_La_3_Si_6_O_20_:Ce^3+^, Tb^3+^ phosphors exhibits broad emission bands corresponding to the allowed *f*–*d* transition of the Ce^3+^ ions and the ^5^D_4_–^7^F_*J*_ characteristic transitions of the Tb^3+^ ions. The emission intensity of the NaBa_3_La_3_Si_6_O_20_:Tb^3+^ samples under excitation wavelength of 268 nm is larger than that under 378 nm, because the intensity of the absorption peak centered at 268 nm is more intense than that at 378 nm. However, the emission light intensity monitored at 268 nm is less than that at 374 nm in the NaBa_3_La_3_Si_6_O_20_:Ce^3+^, Tb^3+^ phosphors. These results verify that it is the overlap between *f*-*f* transition (peaking at 374 nm) but not *f*-*d* transition (peaking at 268 nm) of the Tb^3+^ ions and the emission band of the Ce^3+^ ions induce the energy transfer. Figure 3(b) also shows the excitation spectrum of the NaBa_3_La_3_Si_6_O_20_:0.007Ce^3+^, 0.20Tb^3+^ phosphor monitored at 378 nm (the Ce^3+^ ions emission) is similar to that of at 542 nm (the Tb^3+^ ions emission) except the difference of luminous intensity, which provides another evidence for energy transfer of Ce^3+^ → Tb^3+^.

**Figure 4:**
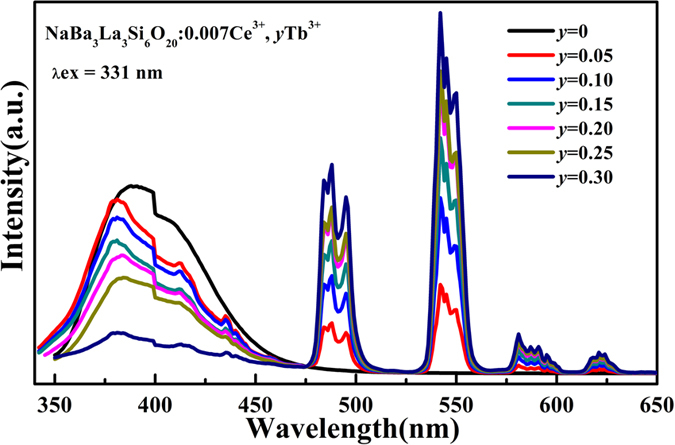
The PL spectra of the NaBa_3_La_3_Si_6_O_20_:0.007Ce^3+^, yTb^3+^ phosphors.

To further investigate the sensitized luminescence of the Tb^3+^ ions by the Ce^3+^ ions, the emission spectra of the NaBa_3_La_3_Si_6_O_20_:0.007Ce^3+^, *y*Tb^3+^ phosphors were measured (Fig. 4). Although the amount of the Ce^3+^ ions is fixed, their emission intensity gradually decreases along with the increase of the concentration of the Tb^3+^ ions. The result indicates that a lot of Tb^3+^ ions as acceptors accelerate energy diffusion of donors, which speeds up the average transfer rate of Ce^3+^ → Tb^3+^.

**Figure 5:**
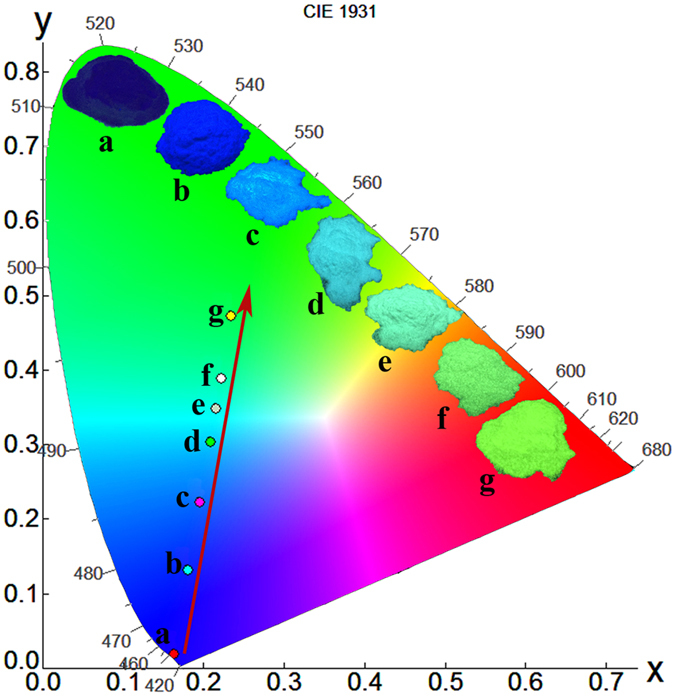
The CIE chromaticity coordinates of the NaBa_3_La_3_Si_6_O_20_:0.007Ce^3+^, yTb^3+^ phosphors (0 ≤ *y* ≤ 0.3) excited under 331 nm and the pictures of the NaBa_3_La_3_Si_6_O_20_:0.007Ce^3+^, *y*Tb^3+^ phosphors (0 ≤ *y* ≤ 0.3) in a 365 nm UV box.

Figure 5 and Table 1 show the variation of Commission International deL’Eclairage (CIE) chromaticity coordinates of the NaBa_3_La_3_Si_6_O_20_:0.007Ce^3+^, *y*Tb^3+^ phosphors (*y* = 0, 0.05, 0.10, 0.15, 0.20, 0.25, 0.30) under excitation wavelength at 331 nm. The insets of Fig. 5 are the photographs of the NaBa_3_La_3_Si_6_O_20_:0.007Ce^3+^, *y*Tb^3+^ phosphors with different amount Tb^3+^ ions in a 365 nm *n*-UV lamp box. These results indicate that the emission light color can be modulated from deep blue to green only by varying the contention of the Tb^3+^ ions. Therefore the NaBa_3_La_3_Si_6_O_20_:Ce^3+^, *y*Tb^3+^ samples can be potential color-tunable phosphors for application in *n*-UV based WLED devices.

### Energy transfer mechanism

In general, the energy transfer from a sensitizer to an activator in oxide may take place via exchange interaction or electric multipolar interaction^48^. The separation distance *R*_Ce–Tb_ can be also estimated from equation (1). Here, *x* is the total concentration of the Ce^3+^ and Tb^3+^ ions, where the luminescence intensity of sensitizer is half of that in samples lack of activator. For the NaBa_3_La_3_Si_6_O_20_:Ce^3+^, Tb^3+^ phosphors, the value of 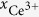 and 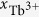 is about 0.021 and 0.75 respectively, thus *R*_Ce–Tb_ is calculated to be about 7.5 Å. Since exchange interaction was restricted to distances of about 4 Å, the energy transfer mechanism of Ce^3+^ → Tb^3+^ should may be electric multipolar interaction^43^,^44^.

**Figure 6:**
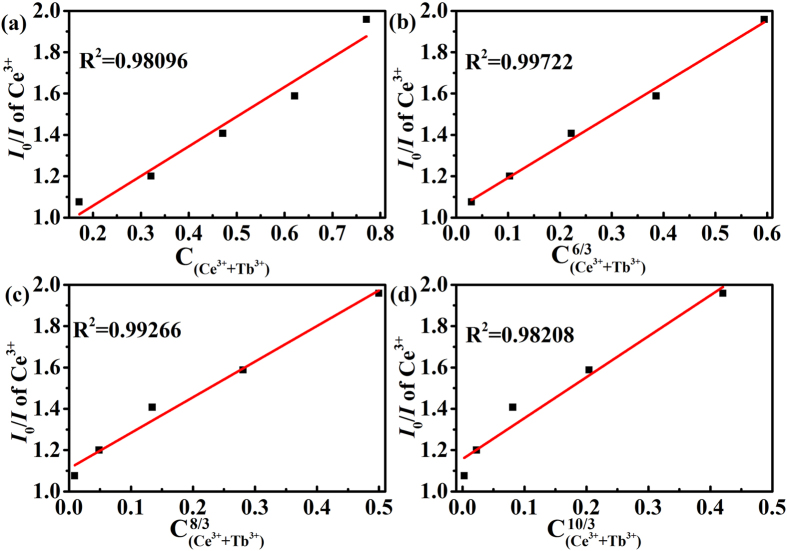
The dependence of *I*_*0*_/*I* of the Ce^3+^ ions on (**a**) 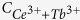 (**b**) 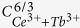 (**c**) 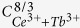 (**d**) 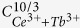.

According to Dexter’s energy transfer expressions of multipolar interaction and Reisfeld’s approximation, the following relation can be given as^42^,^48^,^49^,^50^,^51^:


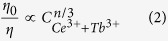


where *η*_*0*_ and *η* are the luminescence quantum efficiency of the Ce^3+^ ions in absence and presence of the Tb^3+^ ions, *n* = 6, 8 and 10 are corresponding to dipole – dipole, dipole – quadrupole and quadrupole – quadrupole interactions, respectively. The value *ƞ*_*0*/ _*ƞ* is approximately estimated by the ratio of related luminescence intensity *I*_*0*_/*I*, *I*_*0*_ is the intrinsic luminescence intensity of the Ce^3+^ ions, and *I* is the luminescence intensity of the Ce^3+^ ions in presence of the Tb^3+^ ions. Figure 6(a–d) illustrates the relationships between *I*_*0*_/*I* and 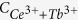 as well as *I*_*0*_/*I* and 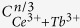. The *R*^2^ value is reasonable in Fig. 6(b,c), implying the energy transfer of Ce^3+^ → Tb^3+^ may occur via dipole–dipole or dipole–quadrupole interaction. However, Sommerdijk stated the probability of energy transfer of Ce^3+^ → Tb^3+^ via electric dipole-dipole interaction was less likely, therefore, dipole – quadrupole interaction should mainly contribute to energy transfer of Ce^3+^ → Tb^3+^ 52.

**Figure 7:**
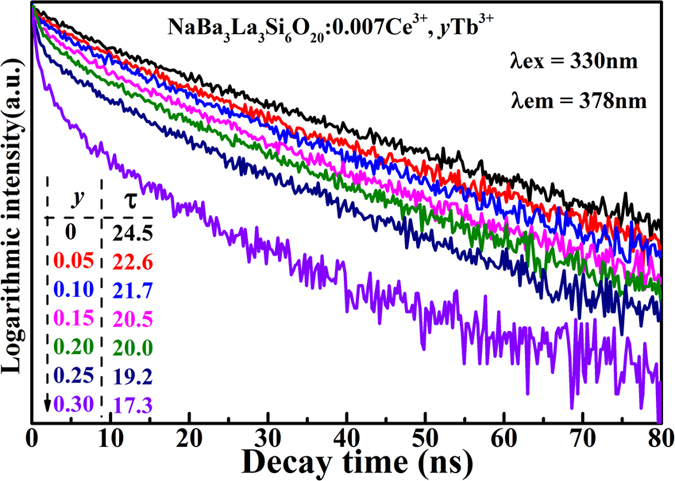
The decay curves for the emission of the Ce^3+^ ions in the NaBa_3_La_3_Si_6_O_20_:0.007Ce^3+^, *y*Tb^3+^ phosphors excited under 330 nm and monitored at 378 nm.

In order to further validate the energy transfer process, the room temperature decay curves for the 4*f*-5*d* (centered at 378 nm) transition of the Ce^3+^ ions in NaBa_3_La_3_Si_6_O_20_:0.007Ce^3+^, *y*Tb^3+^ (*y* = 0.05, 0.10, 0.15, 0.20, 0.25 and 0.30) excited at 330 nm are shown in Fig. 7 For existing two types of Ce^3+^ ions in topic phosphors, the decay curves should be well fitted with a typical two exponential function^53^:


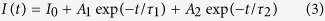


where *I*(*t*) and *I*_0_ are the luminescence intensity at time t, A_1_ and A_2_ are the fitting constants, τ_1_ and τ_2_ represent the decay time for the exponential components. Then the average lifetime (τ*) can be calculated to be 24.5, 22.6, 21.7, 20.5, 20.0, 19.2 and 17.3 by the following formula^40^,^54^:


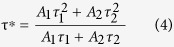


The decay time of the Ce^3+^ ions decreases as increase of the concentration of the Tb^3+^ ions, which strongly demonstrates the energy transfer of Ce^3+^ → Tb^3+^.

**Figure 8:**
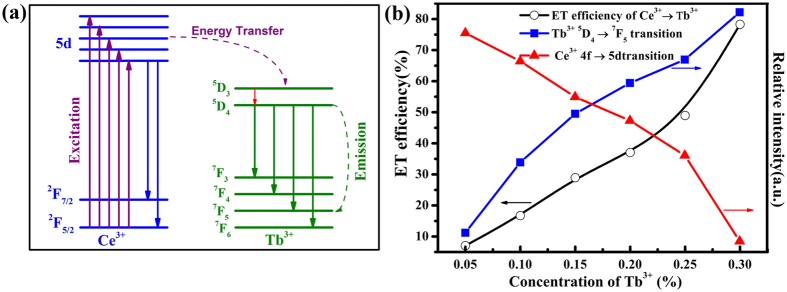
(**a**) The energy levels model for the energy transfer processes of Ce^3+^ → Tb^3+^. (**b**) The dependence of the emission of the Ce^3+^ ions and Tb^3+^ ions, and the energy transfer of Ce^3+^ → Tb^3+^ on the doping concentration of the Tb^3+^ ions in the NaBa_3_La_3_Si_6_O_20_:0.007Ce^3+^, *y*Tb^3+^ phosphors.

Subsequently the energy levels model for the energy transfer processes of Ce^3+^ → Tb^3+^ was investigated. As given in Fig. 8(a), the Ce^3+^ ion absorbs light firstly, then it jumps from the ground states (^2^F_5/2_) to the excited states (5*d* energy levels), subsequently the excited state Ce^3+^ ion returns to the lowest level of 5d levels by giving off excess energy to its surroundings, eventually goes back to the ^2^F_7/2_ or ^2^F_5/2_ ground states by a radiative process. The energy transfer efficiency of Ce^3+^ → Tb^3+^ should increase as the increase of the concentration of the Tb^3+^ ions due to more neighboring Tb^3+^ ions around the Ce^3+^ ions. Finally the energy level transitions of ^5^D_4_ to ^7^F_*J*_ (*J* = 3, 4, 5 and 6) produce the characteristic emission of the Tb^3+^ ions.

The energy transfer efficiency *ƞ*_*T*_ from the Ce^3+^ ions to the Tb^3+^ ions can be calculated according to the following equation^55^:


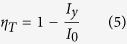


where *I*_*0*_ and *I*_*y*_ are the emission light intensity of the sensitizer with and without an activator, respectively. In the NaBa_3_La_3_Si_6_O_20_:0.007Ce^3+^, yTb^3+^ samples, the Ce^3+^ ion is a sensitizer and the Tb^3+^ ion is an activator. The *ƞ*_*T*_ values can be calculated as 7.06%, 16.71%, 28.95%, 37.04%, 48.95%, 78.31%, as a function of *y* (*y* = 0.15, 0.30, 0.45, 0.60, 0.75, 0.90), respectively (Fig. 8(b)). The energy transfer of Ce^3+^ → Tb^3+^ is consistent with the conclusion that the energy transfer efficiency increases as the increase of the concentration of the Tb^3+^ ions due to more neighboring Tb^3+^ ions around the Ce^3+^ ions and is equivalent to that of the reported K_2_MgSiO_4_:Ce^3+^, Tb^3+^ silicate phosphor^56^.

## Conclusion

A series of novel NaBa_3_La_3_Si_6_O_20_:Ce^3+^, Tb^3+^ phosphors were prepared by solid state method. The energy transfer processe of Ce^3+^ → Tb^3+^ has been demonstrated to be dipole – quadrupole interaction. The tunable colors from deep blue to green can be realized by varying the doping concentration of the Tb^3+^ ions under the irradiation of 331 nm. These results demonstrate the as-prepared NaBa_3_La_3_Si_6_O_20_:Ce^3+^, Tb^3+^ samples can act as potential *n*-UV based w-LED phosphors.

### Experimental Section

#### Compounds synthesis

The NaBa_3_La_3_Si_6_O_20_:Ce^3+^, Tb^3+^ phosphors were synthesized by high temperature solid state method. Na_2_CO_3_ (A.R.), BaCO_3_ (A.R.), SiO_2_ (A.R.), La_2_O_3_ (99.99%), CeO_2_ (99.99%) and Tb_4_O_7_ (99.99%) were purchased from Sinopharm Chemical Reagent Co., Ltd. All of the initial chemicals were used without further purification. Stoichiometric amounts of the above-mentioned chemicals were ground thoroughly by an agate mortar, packed tightly in an alumina crucible. The temperature of the furnace was heated up to 500 °C at a rate of 60 °C/h, then held for 24 h to preheat the mixture in air atmosphere. After the mixture was ground once again, the temperature was increased to 930 °C at a rate of 60 °C/h and held for 100 h with four intermittent grindings. Finally, the prepared phosphors were cooled to room temperature and reground into resulting phosphors. For convenient expression, NaBa_3_La_3−3*x*–3*y*_Ce_3*x*_Tb_3*y*_Si_6_O_20_ is abbreviated as NaBa_3_La_3_Si_6_O_20_:*x*Ce^3+^, *y*Tb^3+^. For example, NaBa_3_La_2.55_Ce_0.15_Tb_0.30_Si_6_O_20_ is denoted as NaBa_3_La_3_Si_6_O_20_:0.05Ce^3+^, 0.10Tb^3+^.

#### Material characterization

The powder XRD measurements were taken on a Bruker D8 X-ray diffractometer with a Cu Kα source (λ = 1.5418 Å) in the angular range from 5° to 80° with a scanning step of 0.15. The structure refinement was carried out with the General Structure Analysis (GSAS) and EXPGUI software^57^,^58^. XRD Rietveld profile refinements of the structural models were performed using the General Structure Analysis (GSAS) software. The photoluminescence (PL) and photoluminescence excitation (PLE) spectra were obtained by an FLS-980 fluorescence spectrophotometer equipped with a 450 W Xe light source. The photoluminescence lifetime curves were measured on an FLS-920 fluorescence spectrophotometer equipped with a laser as light source. All measurements were performed at room temperature. The element analyses of samples were performed by the (X-ray fluorescence) XRF method on a thermo ARL ADVANTXP+ apparatus.

## Additional Information

**How to cite this article**: Jia, Z. and Xia, M. Blue-green tunable color of Ce^3+^/Tb^3+^ coactivated NaBa_3_La_3_Si_6_O_20_ phosphor via energy transfer. *Sci. Rep.* **6**, 33283; doi: 10.1038/srep33283 (2016).

## Supplementary Material

Supplementary Information

## Figures and Tables

**Table 1:** The comparison of the CIE chromaticity coordinates of the NaBa_3_La_3_Si_6_O_20_:0.007Ce^3+^, yTb^3+^ phosphors (λ_ex_ = 331 nm).

No. of points in CIE diagram	Sample compositions NaBa_3_La_3_Si_6_O_20_:0.007Ce^3+^, yTb^3+^	CIE coordinates (*x*, *y*)
a	y = 0.00	(0.163, 0.019)
b	y = 0.05	(0.181, 0.131)
c	y = 0.10	(0.195, 0.222)
d	y = 0.15	(0.208, 0.303)
e	y = 0.20	(0.216, 0.348)
f	y = 0.25	(0.223, 0.389)
g	y = 0.30	(0.235, 0.472)
