# Supplementary material for: Blue-green tunable color of Ce3+/Tb3+ coactivated NaBa3La3Si6O20 phosphor via energy transfer
Source: Sci Rep. 2016 Sep 15;6:33283. doi: 10.1038/srep33283 (PMC5024107; doi:10.1038/srep33283)
Supplement: Supplementary Information [file srep33283-s1.pdf]

Supplementary Information

Blue-green tunable color of Ce^{3+}/Tb^{3+} coactivated $NaBa_3La_3Si_6O_{20}$ phosphor via energy transfer

Zhen Jia[1,2,3] & Mingjun Xia[2,*]

[1] Key Laboratory of Coordination Chemistry and Functional Materials in Universities of Shandong, Dezhou University, Dezhou 253023, PR China.

[2] Beijing Center for Crystal Research and Development, Key Laboratory of Functional Crystals and Laser Technology, Technical Institute of Physics and Chemistry, Chinese Academy of Sciences, Beijing 100190, PR China.

[3] University of Chinese Academy of Sciences, Beijing 100049, PR China.

*Corresponding author: M. J. X (Email: xiamingjun@mail.ipc.ac.cn)

Supplementary Tables:

Table S1. Atomic percentage of $NaBa_3La_{3(1-x-y)}Ce_{3x}Tb_{3y}Si_6O_{20}$ phosphors measured by X-ray fluorescence method on a Thermo ARL ADVANTXP+ apparatus.

Table S2. The coordination, occupancy and isotropic displacement parameter for $NaBa_3La_3Si_6O_{20}$.

Table S3. The coordination, occupancy and isotropic displacement parameter for $NaBa_3La_3Si_6O_{20}$:0.007Ce^{3+}.

Table S4. The coordination, occupancy and isotropic displacement parameter for $NaBa_3La_3Si_6O_{20}$:0.007Ce^{3+},0.05Tb^{3+}.

Table S5. The coordination, occupancy and isotropic displacement parameter for $NaBa_3La_3Si_6O_{20}$:0.007Ce^{3+},0.10Tb^{3+}.

Table S6. The coordination, occupancy and isotropic displacement parameter for $NaBa_3La_3Si_6O_{20}$:0.007Ce^{3+},0.15Tb^{3+}.

Table S7. The coordination, occupancy and isotropic displacement parameter for $NaBa_3La_3Si_6O_{20}$:0.007Ce^{3+},0.20Tb^{3+}.

Table S8. The coordination, occupancy and isotropic displacement parameter for $NaBa_3La_3Si_6O_{20}$:0.007Ce^{3+},0.25Tb^{3+}.

Table S9. The coordination, occupancy and isotropic displacement parameter for $NaBa_3La_3Si_6O_{20}$:0.007Ce^{3+},0.30Tb^{3+}.

Supplementary Figures:

Figure S1: The XRD profiles for the Rietveld refinement of $NaBa_3La_3Si_6O_{20}$:0.007Ce^{3+}.

Figure S2. The XRD profiles for the Rietveld refinement of $NaBa_3La_3Si_6O_{20}$:0.007Ce^{3+},0.05Tb^{3+}.

Figure S3. The XRD profiles for the Rietveld refinement of $NaBa_3La_3Si_6O_{20}$:0.007Ce^{3+},0.10Tb^{3+}.

Figure S4. The XRD profiles for the Rietveld refinement of $NaBa_3La_3Si_6O_{20}$:0.007Ce^{3+},0.15Tb^{3+}.

Figure S5. The XRD profiles for the Rietveld refinement of $NaBa_3La_3Si_6O_{20}$:0.007Ce^{3+},0.20Tb^{3+}.

Figure S6. The XRD profiles for the Rietveld refinement of $NaBa_3La_3Si_6O_{20}$:0.007Ce^{3+},0.25Tb^{3+}.

Figure S7. The XRD profiles for the Rietveld refinement of $NaBa_3La_3Si_6O_{20}$:0.007Ce^{3+},0.30Tb^{3+}

Table S1. Atomic percentage of $NaBa_3La_{3(1-x-y)}Ce_{3x}Tb_{3y}Si_6O_{20}$ phosphors measured by X-ray fluorescence method on a Thermo ARL ADVANTXP+ apparatus.

Sample compositions of $NaBa_3La_{3(1-x-y)}Ce_{3x}Tb_{3y}Si_6O_{20}$	Na	Si	Ba	La	Ce	Tb
x = 0, y=0	7.25	15.77	32.21	44.77	0	0
x = 0.007, y = 0	7.23	15.97	32.12	44.30	0.38	0
x = 0.007, y = 0.05	6.78	15.34	32.59	42.34	0.37	2.58
x = 0.007, y = 0.10	7.27	15.81	32.53	39.34	0.32	4.73
x = 0.007, y = 0.15	7.01	15.90	31.53	37.75	0.33	7.48
x = 0.007, y = 0.20	6.49	15.35	32.78	35.33	0.31	9.74
x = 0.007, y = 0.25	6.88	15.47	32.17	32.84	0.33	12.31
x = 0.007, y = 0.30	7.13	15.66	32.31	29.98	0.34	14.58

Table S2. The coordination, occupancy and isotropic displacement parameter for $NaBa_3La_3Si_6O_{20}$.

Atoms	x	y	z	Occupancy	Uiso
Ba1	0.75	0.9171	0.0872	1	0.002
Ba2	0.3883	0.7443	0.5972	1	0.002
La1	0.5321	0.8868	0.5337	1	0.004
La2	0.5	0	0.0213	1	0.003
Na1	0.75	0.1159	0.7030	1	0.03
Si1	0.4406	0.8486	0.0606	1	0.009
Si2	0.75	0.8178	0.5530	1	0.009
Si3	0.6391	0.9986	0.5839	1	0.009
Si4	0.75	0.6884	0.6112	1	0.009
O1	0.3351	0.8417	-0.0091	1	0.02
O2	0.4682	0.8105	0.2961	1	0.02
O3	0.4971	0.8271	-0.1709	1	0.02
O4	0.4655	0.9116	0.1437	1	0.02
O5	0.75	0.7516	0.4900	1	0.02
O6	0.6618	0.8456	0.4229	1	0.02
O7	0.75	0.8243	0.8500	1	0.02
O8	0.75	0.0125	0.5770	1	0.02
O9	0.6166	0.9522	0.7854	1	0.02
O10	0.5876	0.0553	0.6641	1	0.02
O11	0.6069	0.9733	0.3310	1	0.02
O12	0.25	0.8091	0.4025	1	0.02

a = 14.9226(4) Å, b = 24.5215(5) Å and c = 5.6241(2) Å

Table S3. The coordination, occupancy and isotropic displacement parameter for $NaBa_3La_3Si_6O_{20}$:$0.007Ce^{3+}$.

Atoms	x	y	z	Occupancy	Uiso
Ba1	0.75	0.9183	0.0958	1	0.002
Ba2	0.3874	0.7441	0.5981	1	0.002
La1	0.5301	0.8877	0.5382	0.993	0.004
Ce1	0.5301	0.8877	0.5382	0.007	0.004
La2	0.5	0	0.0238	0.993	0.003
Ce2	0.5	0	0.0238	0.007	0.003
Na1	0.75	0.0997	0.6756	1	0.03
Si1	0.4423	0.8487	0.0606	1	0.009
Si2	0.75	0.8199	0.5630	1	0.009
Si3	0.6399	0.9997	0.5838	1	0.009
Si4	0.75	0.6901	0.6166	1	0.009
O1	0.3351	0.8417	-0.0091	1	0.02
O2	0.4682	0.8105	0.2961	1	0.02
O3	0.4971	0.8271	-0.1709	1	0.02
O4	0.4655	0.9116	0.1437	1	0.02
O5	0.75	0.7516	0.4900	1	0.02
O6	0.6618	0.8456	0.4229	1	0.02
O7	0.75	0.8243	0.8500	1	0.02
O8	0.75	0.0125	0.5770	1	0.02
O9	0.6166	0.9522	0.7854	1	0.02
O10	0.5876	0.0553	0.6641	1	0.02
O11	0.6069	0.9733	0.3310	1	0.02
O12	0.25	0.8091	0.4025	1	0.02

a = 14.9102(5) Å, b = 24.5158(8) Å and c = 5.6155(2) Å

Table S4. The coordination, occupancy and isotropic displacement parameter for $NaBa_3La_3Si_6O_{20}$:0.007Ce^{3+},0.05Tb^{3+}.

Atoms	x	y	z	Occupancy	Uiso
Ba1	0.75	0.9181	0.0952	1	0.002
Ba2	0.3876	0.7453	0.5921	1	0.002
La1	0.5309	0.8869	0.5285	0.943	0.004
Ce1	0.5309	0.8869	0.5285	0.007	0.004
Tb1	0.5309	0.8869	0.5285	0.05	0.004
La2	0.5	0	0.0274	0.943	0.003
Ce1	0.5	0	0.0274	0.007	0.003
Tb1	0.5	0	0.0274	0.05	0.003
Na1	0.75	0.1110	0.6661	1	0.03
Si1	0.4423	0.8487	0.0606	1	0.009
Si2	0.75	0.8199	0.5630	1	0.009
Si3	0.6399	0.9997	0.5838	1	0.009
Si4	0.75	0.6901	0.6166	1	0.009
O1	0.3351	0.8417	-0.0091	1	0.02
O2	0.4682	0.8105	0.2961	1	0.02
O3	0.4971	0.8271	-0.1709	1	0.02
O4	0.4655	0.9116	0.1437	1	0.02
O5	0.75	0.7516	0.4900	1	0.02
O6	0.6618	0.8456	0.4229	1	0.02
O7	0.75	0.8243	0.8500	1	0.02
O8	0.75	0.0125	0.5770	1	0.02
O9	0.6166	0.9522	0.7854	1	0.02
O10	0.5876	0.0553	0.6641	1	0.02
O11	0.6069	0.9733	0.3310	1	0.02
O12	0.25	0.8091	0.4025	1	0.02

a = 14.9076(5) Å, b = 24.4845(8) Å and c = 5.6213(2) Å

Table S5. The coordination, occupancy and isotropic displacement parameter for $NaBa_3La_3Si_6O_{20}$:0.007Ce^{3+},0.10Tb^{3+}.

Atoms	x	y	z	Occupancy	Uiso
Ba1	0.75	0.9173	0.0956	1	0.002
Ba2	0.3877	0.7458	0.5942	1	0.002
La1	0.5314	0.8869	0.5344	0.893	0.004
Ce1	0.5314	0.8869	0.5344	0.007	0.004
Tb1	0.5314	0.8869	0.5344	0.1	0.004
La2	0.5	0	0.0251	0.893	0.003
Ce1	0.5	0	0.0251	0.007	0.003
Tb1	0.5	0	0.0251	0.1	0.003
Na1	0.75	0.1159	0.7030	1	0.03
Si1	0.4423	0.8487	0.0606	1	0.009
Si2	0.75	0.8199	0.5630	1	0.009
Si3	0.6399	0.9997	0.5838	1	0.009
Si4	0.75	0.6901	0.6166	1	0.009
O1	0.3351	0.8417	-0.0091	1	0.02
O2	0.4682	0.8105	0.2961	1	0.02
O3	0.4971	0.8271	-0.1709	1	0.02
O4	0.4655	0.9116	0.1437	1	0.02
O5	0.75	0.7516	0.4900	1	0.02
O6	0.6618	0.8456	0.4229	1	0.02
O7	0.75	0.8243	0.8500	1	0.02
O8	0.75	0.0125	0.5770	1	0.02
O9	0.6166	0.9522	0.7854	1	0.02
O10	0.5876	0.0553	0.6641	1	0.02
O11	0.6069	0.9733	0.3310	1	0.02
O12	0.25	0.8091	0.4025	1	0.02

a = 14.8914(5) Å, b = 24.4478(8) Å and c = 5.6174(2) Å

Table S6. The coordination, occupancy and isotropic displacement parameter for $NaBa_3La_3Si_6O_{20}$:0.007Ce^{3+},0.15Tb^{3+}.

Atoms	x	y	z	Occupancy	Uiso
Ba1	0.75	0.9194	0.0941	1	0.002
Ba2	0.3862	0.7431	0.5941	1	0.002
La1	0.5306	0.8894	0.5217	0.843	0.004
Ce1	0.5306	0.8894	0.5217	0.007	0.004
Tb1	0.5306	0.8894	0.5217	0.15	0.004
La2	0.5	0	0.0218	0.843	0.003
Ce1	0.5	0	0.0218	0.007	0.003
La1	0.5	0	0.0218	0.15	0.003
Na1	0.75	0.1110	0.6661	1	0.03
Si1	0.4423	0.8487	0.0606	1	0.009
Si2	0.75	0.8199	0.5630	1	0.009
Si3	0.6399	0.9997	0.5838	1	0.009
Si4	0.75	0.6901	0.6166	1	0.009
O1	0.3351	0.8417	-0.0091	1	0.02
O2	0.4682	0.8105	0.2961	1	0.02
O3	0.4971	0.8271	-0.1709	1	0.02
O4	0.4655	0.9116	0.1437	1	0.02
O5	0.75	0.7516	0.4900	1	0.02
O6	0.6618	0.8456	0.4229	1	0.02
O7	0.75	0.8243	0.8500	1	0.02
O8	0.75	0.0125	0.5770	1	0.02
O9	0.6166	0.9522	0.7854	1	0.02
O10	0.5876	0.0553	0.6641	1	0.02
O11	0.6069	0.9733	0.3310	1	0.02
O12	0.25	0.8091	0.4025	1	0.02

a = 14.8684(5) Å, b = 24.4045(2) Å and c = 5.6121(3) Å

Table S7. The coordination, occupancy and isotropic displacement parameter for $NaBa_3La_3Si_6O_{20}$:0.007Ce^{3+},0.20Tb^{3+}.

Atoms	x	y	z	Occupancy	Uiso
Ba1	0.75	0.9166	0.0857	1	0.002
Ba2	0.3867	0.7447	0.5881	1	0.002
La1	0.5324	0.8873	0.5222	0.793	0.004
Ce1	0.5324	0.8873	0.5222	0.007	0.004
Tb1	0.5324	0.8873	0.5222	0.2	0.004
La2	0.5	0	0.0212	0.793	0.003
Ce1	0.5	0	0.0212	0.007	0.003
Tb1	0.5	0	0.0212	0.2	0.003
Na1	0.75	0.1251	0.6623	1	0.03
Si1	0.4423	0.8487	0.0606	1	0.009
Si2	0.75	0.8199	0.5630	1	0.009
Si3	0.6399	0.9997	0.5838	1	0.009
Si4	0.75	0.6901	0.6166	1	0.009
O1	0.3351	0.8417	-0.0091	1	0.02
O2	0.4682	0.8105	0.2961	1	0.02
O3	0.4971	0.8271	-0.1709	1	0.02
O4	0.4655	0.9116	0.1437	1	0.02
O5	0.75	0.7516	0.4900	1	0.02
O6	0.6618	0.8456	0.4229	1	0.02
O7	0.75	0.8243	0.8500	1	0.02
O8	0.75	0.0125	0.5770	1	0.02
O9	0.6166	0.9522	0.7854	1	0.02
O10	0.5876	0.0553	0.6641	1	0.02
O11	0.6069	0.9733	0.3310	1	0.02
O12	0.25	0.8091	0.4025	1	0.02

a = 14.8542(5) Å, b = 24.3734(8) Å and c = 5.6088(2) Å

Table S8. The coordination, occupancy and isotropic displacement parameter for $NaBa_3La_3Si_6O_{20}$:0.007Ce^{3+},0.25Tb^{3+}.

Atoms	x	y	z	Occupancy	Uiso
Ba1	0.75	0.9194	0.0941	1	0.002
Ba2	0.3862	0.7431	0.5941	1	0.002
La1	0.5306	0.8894	0.5217	0.743	0.004
Ce1	0.5306	0.8894	0.5217	0.007	0.004
Tb1	0.5306	0.8894	0.5217	0.25	0.004
La2	0.5	0	0.0217	0.743	0.003
Ce1	0.5	0	0.0217	0.007	0.003
Tb1	0.5	0	0.0217	0.25	0.003
Na1	0.75	0.1257	0.6707	1	0.03
Si1	0.4423	0.8487	0.0606	1	0.009
Si2	0.75	0.8199	0.5630	1	0.009
Si3	0.6399	0.9997	0.5838	1	0.009
Si4	0.75	0.6901	0.6166	1	0.009
O1	0.3351	0.8417	-0.0091	1	0.02
O2	0.4682	0.8105	0.2961	1	0.02
O3	0.4971	0.8271	-0.1709	1	0.02
O4	0.4655	0.9116	0.1437	1	0.02
O5	0.75	0.7516	0.4900	1	0.02
O6	0.6618	0.8456	0.4229	1	0.02
O7	0.75	0.8243	0.8500	1	0.02
O8	0.75	0.0125	0.5770	1	0.02
O9	0.6166	0.9522	0.7854	1	0.02
O10	0.5876	0.0553	0.6641	1	0.02
O11	0.6069	0.9733	0.3310	1	0.02
O12	0.25	0.8091	0.4025	1	0.02

a = 14.8398(9) Å, b = 24.3379(2) Å and c = 5.6050(3) Å

Table S9. The coordination, occupancy and isotropic displacement parameter for $NaBa_3La_3Si_6O_{20}$:0.007Ce^{3+},0.30Tb^{3+}.

Atoms	x	y	z	Occupancy	Uiso
Ba1	0.75	0.9161	0.0886	1	0.002
Ba2	0.3863	0.7439	0.5904	1	0.002
La1	0.5307	0.8878	0.5239	0.693	0.004
Ce1	0.5307	0.8878	0.5239	0.007	0.004
Tb1	0.5307	0.8878	0.5239	0.3	0.004
La2	0.5	0	0.0164	0.693	0.003
Ce1	0.5	0	0.0164	0.007	0.003
Tb1	0.5	0	0.0164	0.3	0.003
Na1	0.75	0.1138	0.6729	1	0.03
Si1	0.4423	0.8487	0.0606	1	0.009
Si2	0.75	0.8199	0.5630	1	0.009
Si3	0.6399	0.9997	0.5838	1	0.009
Si4	0.75	0.6901	0.6166	1	0.009
O1	0.3351	0.8417	-0.0091	1	0.02
O2	0.4682	0.8105	0.2961	1	0.02
O3	0.4971	0.8271	-0.1709	1	0.02
O4	0.4655	0.9116	0.1437	1	0.02
O5	0.75	0.7516	0.4900	1	0.02
O6	0.6618	0.8456	0.4229	1	0.02
O7	0.75	0.8243	0.8500	1	0.02
O8	0.75	0.0125	0.5770	1	0.02
O9	0.6166	0.9522	0.7854	1	0.02
O10	0.5876	0.0553	0.6641	1	0.02
O11	0.6069	0.9733	0.3310	1	0.02
O12	0.25	0.8091	0.4025	1	0.02

a = 14.8271(7) Å, b = 24.3014(8) Å and c = 5.5998(2) Å

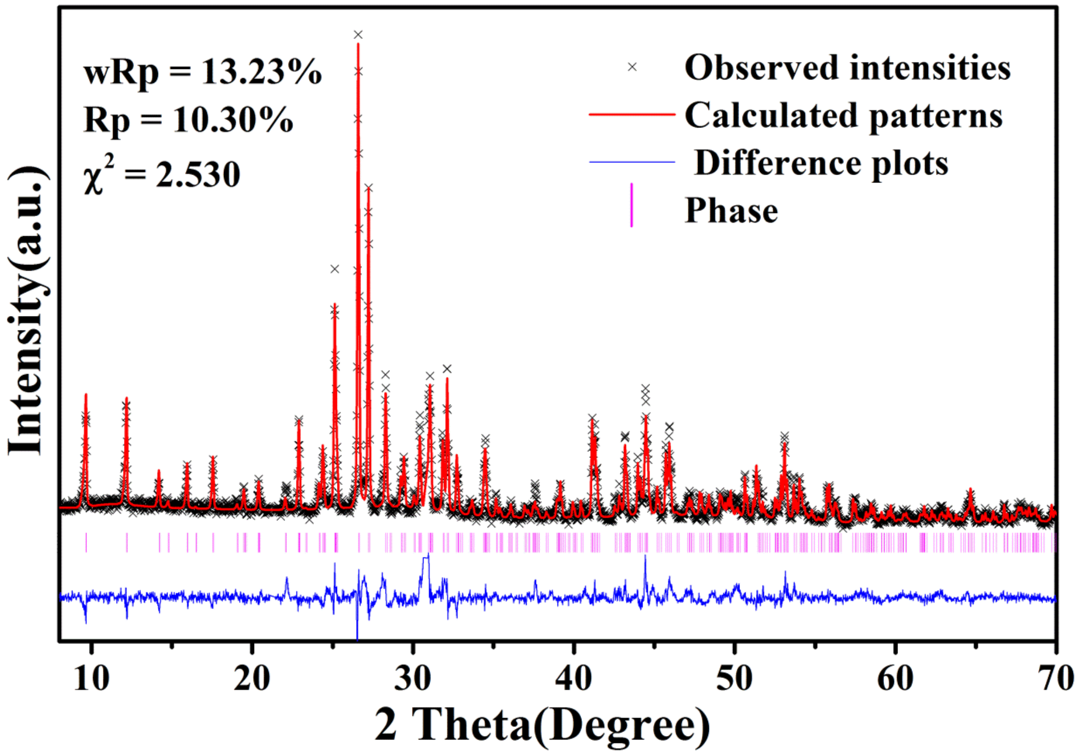

Figure S1. The XRD profiles for the Rietveld refinement of $NaBa_3La_3Si_6O_{20}$:0.007Ce^{3+}.

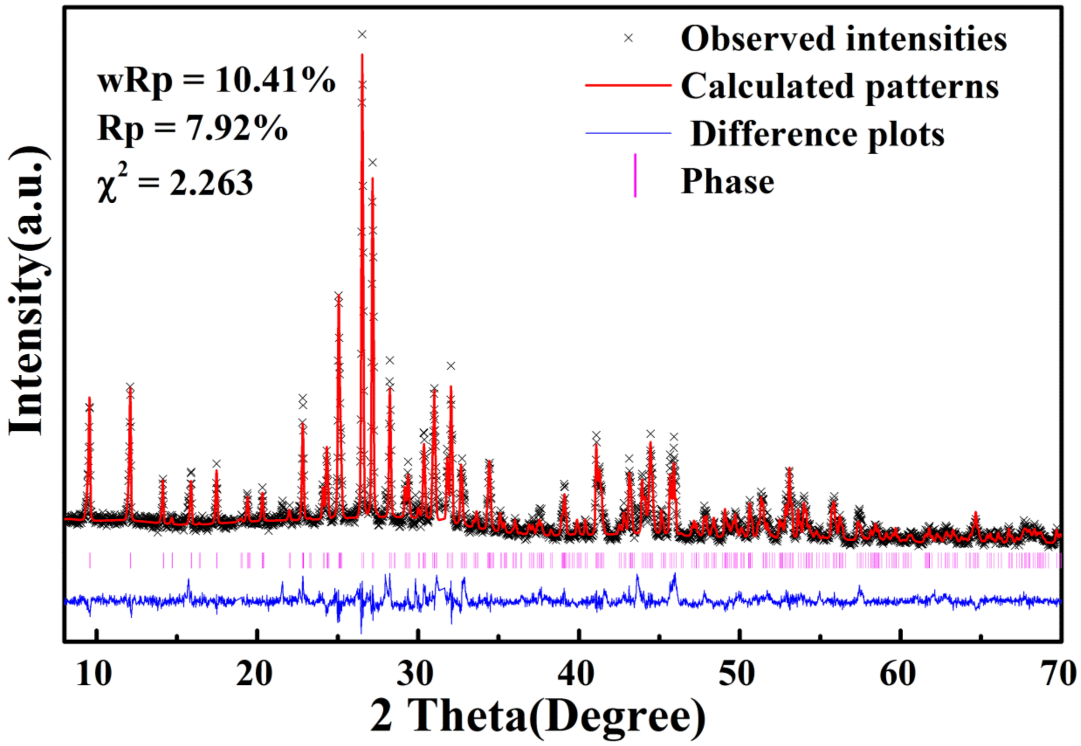

Figure S2. The XRD profiles for the Rietveld refinement of $NaBa_3La_3Si_6O_{20}$:0.007Ce^{3+},0.05Tb^{3+}.

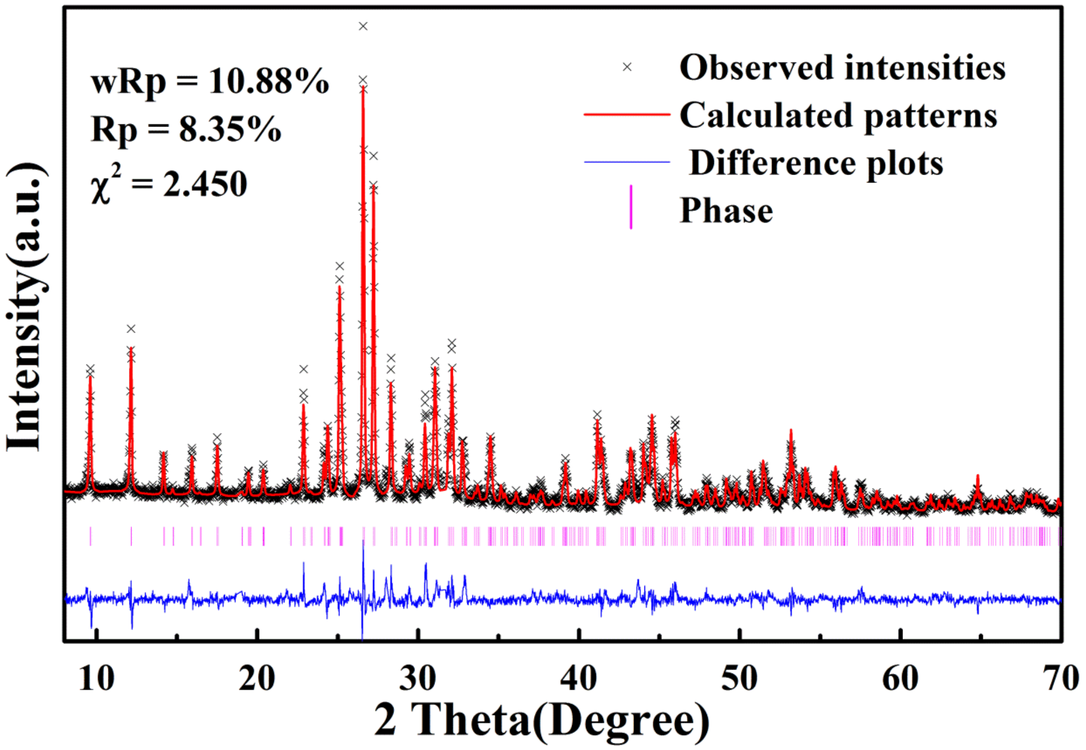

Figure S3. The XRD profiles for the Rietveld refinement of $NaBa_3La_3Si_6O_{20}$:0.007Ce^{3+},0.10Tb^{3+}.

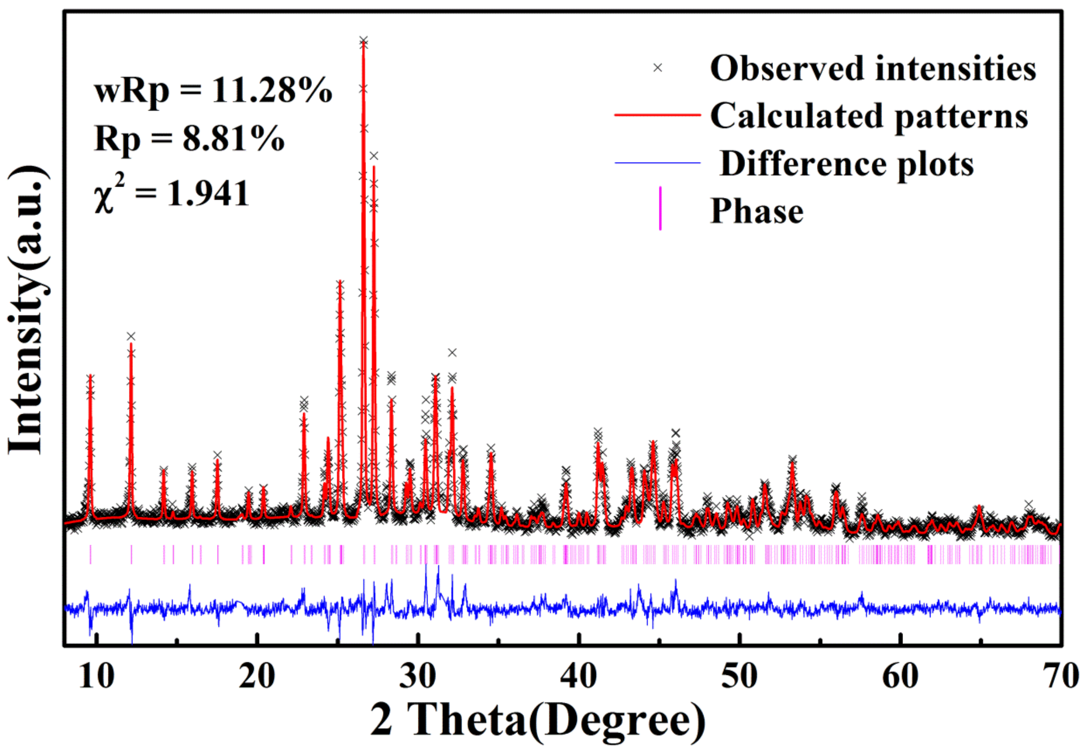

Figure S4. The XRD profiles for the Rietveld refinement of $NaBa_3La_3Si_6O_{20}$:0.007Ce^{3+},0.15Tb^{3+}.

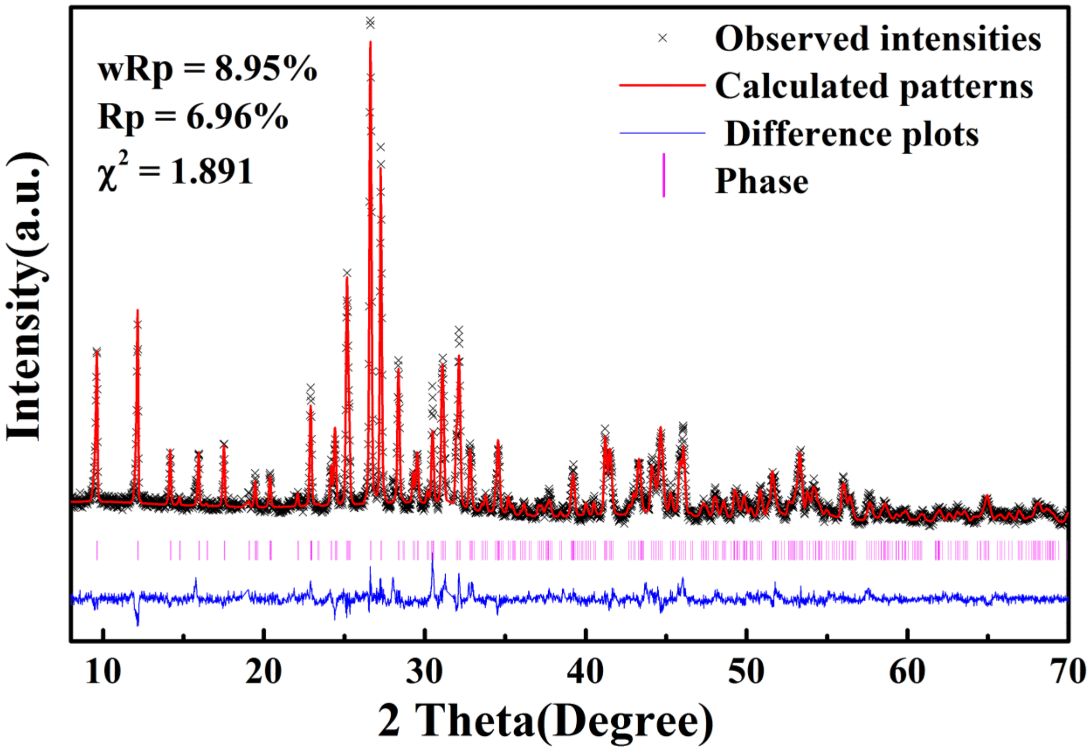

Figure S5. The XRD profiles for the Rietveld refinement of $NaBa_3La_3Si_6O_{20}$:0.007Ce^{3+},0.20Tb^{3+}.

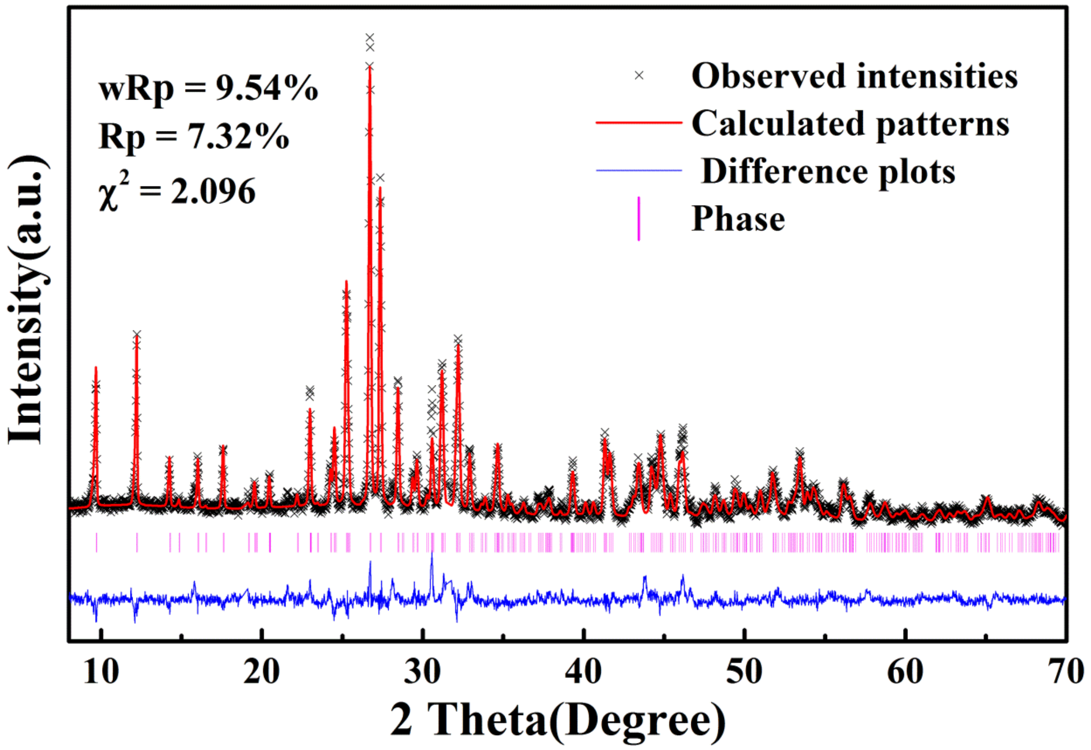

Figure S6. The XRD profiles for the Rietveld refinement of $NaBa_3La_3Si_6O_{20}$:0.007Ce^{3+},0.25Tb^{3+}.

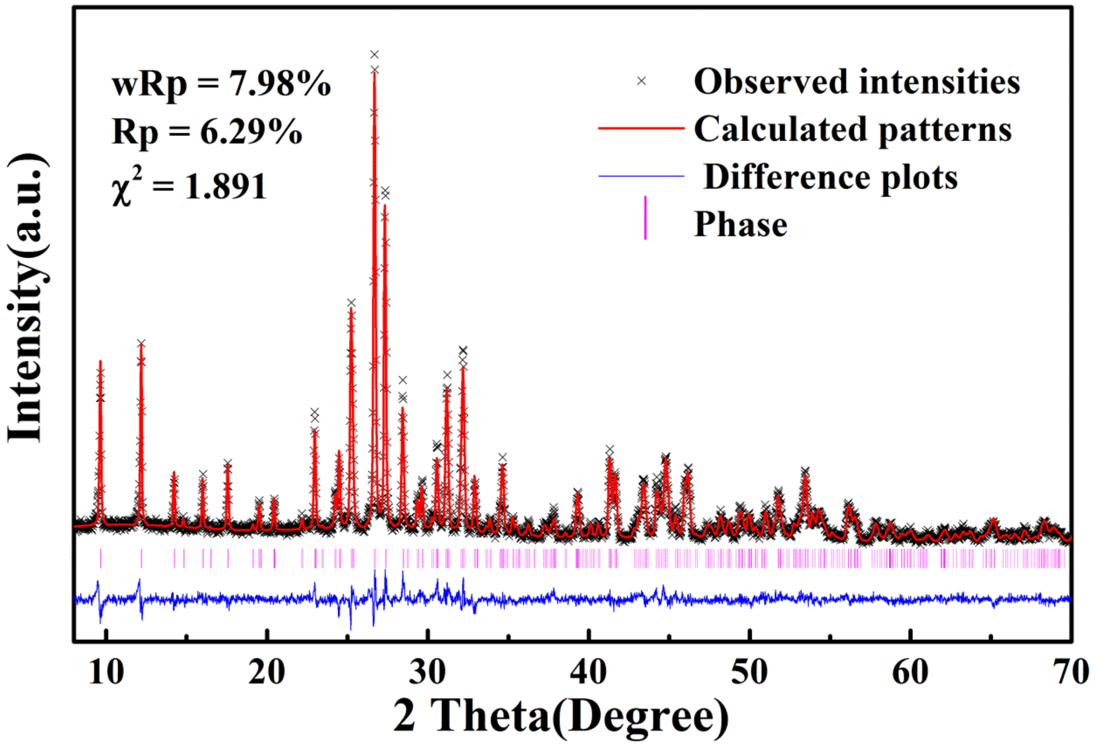

Figure S7. The XRD profiles for the Rietveld refinement of $NaBa_3La_3Si_6O_{20}$:0.007Ce^{3+},0.30Tb^{3+}.
